# The Cerebral Blood Flow Biomedical Informatics Research Network (CBFBIRN) database and analysis pipeline for arterial spin labeling MRI data

**DOI:** 10.3389/fninf.2013.00021

**Published:** 2013-10-18

**Authors:** David D. Shin, I. Burak Ozyurt, Thomas T. Liu

**Affiliations:** ^1^Center for Functional Magnetic Resonance Imaging, University of California at San DiegoLa Jolla, CA, USA; ^2^Department of Psychiatry, University of California at San DiegoLa Jolla, CA, USA

**Keywords:** database, data sharing, arterial spin labeling, cerebral blood flow, perfusion imaging, group analysis, magnetic resonance imaging

## Abstract

Arterial spin labeling (ASL) is a magnetic resonance imaging technique that provides a non-invasive and quantitative measure of cerebral blood flow (CBF). After more than a decade of active research, ASL is now emerging as a robust and reliable CBF measurement technique with increased availability and ease of use. There is a growing number of research and clinical sites using ASL for neuroscience research and clinical care. In this paper, we present an online CBF Database and Analysis Pipeline, collectively called the Cerebral Blood Flow Biomedical Informatics Research Network (CBFBIRN) that allows researchers to upload and share ASL and clinical data. In addition to serving the role as a central data repository, the CBFBIRN provides a streamlined data processing infrastructure for CBF quantification and group analysis, which has the potential to accelerate the discovery of new scientific and clinical knowledge. All capabilities and features built into the CBFBIRN are accessed online using a web browser through a secure login. In this work, we begin with a general description of the CBFBIRN system data model and its architecture, then devote the remainder of the paper to the CBFBIRN capabilities. The latter part of our work is divided into two processing modules: (1) Data Upload and CBF Quantification Module; (2) Group Analysis Module that supports three types of analysis commonly used in neuroscience research. To date, the CBFBIRN hosts CBF maps and associated clinical data from more than 1,300 individual subjects. The data have been contributed by more than 20 different research studies, investigating the effect of various conditions on CBF including Alzheimer’s, schizophrenia, bipolar disorder, depression, traumatic brain injury, HIV, caffeine usage, and methamphetamine abuse. Several example results, generated by the CBFBIRN processing modules, are presented. We conclude with the lessons learned during implementation and deployment of the CBFBIRN and our experience in promoting data sharing.

## INTRODUCTION

Arterial spin labeling (ASL) is a magnetic resonance imaging (MRI) technique (Detre et al., 1992) that provides a quantitative measure of cerebral blood flow (CBF). Over the past decade, ASL has emerged as a robust and non-invasive method for acquiring a regional CBF map with whole-brain coverage in less than 5 minutes on commercial MRI scanners. A growing number of research and clinical sites (Deibler et al., 2008; Pollock et al., 2009) are now using ASL as part of their imaging protocols, and are collectively creating a rich and diverse set of CBF data. The availability of these existing data combined with an increasing number of new data sets underscore the potential benefits of having a central CBF database that enables investigators to upload, analyze, explore, and share ASL data.

There has been an increased awareness for data sharing in the neuroimaging community and the practice of sharing is growing (Van Horn and Toga, 2009; Poline et al., 2012). The Functional Biomedical Research Network (FBIRN) provides an example of successful data sharing through careful coordination on the part of the participating institutions, leading to advances in our understanding of schizophrenia (Potkin and Ford, 2009; Potkin et al., 2009; Glover et al., 2012; Shin et al., 2013). The efficient sharing of neuroimaging and clinical data collected at each institution was possible through the development of a federated database system and software tools (Keator et al., 2009; Ozyurt et al., 2010).

In this paper, we present a central CBF database and associated data analysis workflows, collectively called the Cerebral Blood Flow Biomedical Informatics Research Network (CBFBIRN). The overall goal of the CBFBIRN is to support the interaction of the CBF database with the neuroimaging community, to facilitate data sharing, and to promote collaborative research environment for the study of CBF measures. The CBFBIRN database architecture is based on the Human Imaging Database (HID) framework (Ozyurt et al., 2010), initially developed and used under the FBIRN as a federated database system. The CBFBIRN serves not only as a central data repository for ASL data and associated clinical assessments, but it also provides streamlined processing workflows that allow users to perform CBF processing on the raw data and group analysis on the derived data through a web browser^1^. Additionally, the CBFBIRN hosts a dedicated pulse sequence distribution system (PSDS), through which pulsed arterial spin labeling (PASL) and pseudocontinuous arterial spin labeling (PCASL) protocols are provided to the neuroimaging community. The PASL protocol distributed via the PSDS uses a standardized flow-sensitive alternating inversion recovery (FAIR) sequence with QUIPPSS II post-inversion saturation pulses (Kim and Tsekos, 1997; Wong et al., 1998) and it is the same protocol that was used in previous multisite FBIRN studies (East Coast Traveling Study, West Coast Traveling Study, Phase III schizophrenic vs. healthy control study; Liu et al., 2008; Ford et al., 2009; Rasmussen et al., 2010). The ASL data generated by these FBIRN studies, as well as associated clinical assessments, have been aggregated, archived, and processed by the CBFBIRN.

Within the ASL community, data sharing through a central CBF database is beneficial for several reasons. One main hindrance to a wider adoption of ASL by the neuroimaging community is a lack of standardized data acquisition and processing methods. Pooling of ASL data across different sites into a central repository facilitates a careful evaluation of different acquisition and post processing methods, resulting in (1) improved understanding of how different methods affect the accuracy and reliability of quantified CBF measures; (2) formulation of a standard ASL protocol and a common CBF quantification method that can minimize inter-site differences and promote a faster adoption of ASL. Additionally, a large volume of data allows efficient testing of new processing algorithms and their comparison with preexisting ones. Perhaps more importantly, a central CBF database can greatly facilitate efforts to characterize the dependence of CBF on disease, age, gender, and medical treatment using statistical analysis on the archived CBF data and associated clinical assessments. A large volume of pooled data provides greater statistical power to detect subtle effects that may otherwise be impossible to discern with a limited sample size.

The objectives of this paper are to describe the currently available features and capabilities of the CBFBIRN and to show example results processed from the ASL data that have been contributed by CBFBIRN users. While the CBFBIRN is built for storing, processing, and sharing of ASL data, the system framework can be extended to handle virtually all types of scientific data and thus can be a useful resource for the neuroinformatics community. We provide the implementation details with access to the source code underlying the CBFBIRN system as well as some discussion regarding the lessons learned during the system deployment and our experience in promoting data sharing.

## MATERIALS AND METHODS

### SYSTEM DATA MODEL

The HID framework (Ozyurt et al., 2010) on which the CBFBIRN is built uses an Entity–Attribute–Value (EAV) style database design (Nadkarni et al., 1999; Anhoj, 2003; Marenco et al., 2003) allowing system extension without database schema changes. System extension is achieved by storing both data and metadata in the same schema. In traditional databases, a new concept is added to the database schema using one or more tables. However, the challenge of managing scientific data comes from its complex and constantly changing structure. By identifying the stationary concepts which are common in neuroscientific experiments and separating the abstract concept and its multiple realizations, the HID schema is able to stay stable while accommodating all types of data stored, processed, and generated by the CBFBIRN, i.e., experiment/study attributes, subject demographics, clinical assessments, provenance and raw/derived data. For the CBFBIRN, relatively small extensions to the database schema are needed, i.e., to enable post processing workflow management, raw data upload capability, job status and data summary reporting, provenance data management, security and quality assurance functionality. In this paper, only the additional changes from the HID framework are described. The core data model is described in the original publication (Ozyurt et al., 2010).

**FIGURE 1:**
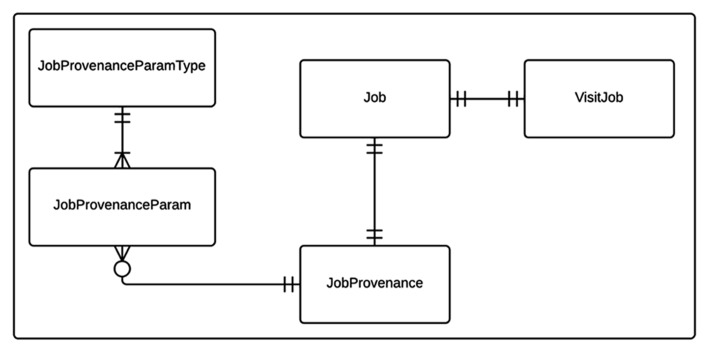
**Entity relationship (ER) diagram of CBFBIRN processing workflow data model.** The relationships between the entities (database tables) including cardinality information are further explained in the text.

Cerebral blood flow processing and group analysis workflows with their corresponding provenance data are modeled as shown in **Figure 1**. The state relevant for a workflow instance is stored in a Job table. Each CBF processing job instance is associated with a corresponding imaging visit (VisitJob). Unlike a CBF processing job, a group analysis workflow instance can be associated with one or more imaging visits, which may come from multiple experiments/projects. Each workflow instance (job) in turn is associated with a JobProvenance mainly acting as a grouping mechanism for a set of JobProvenanceParam records encoding provenance data of the corresponding workflow instance. The JobProvenance record also points to the local file path on the database where all the derived data from a corresponding CBF processing or a group analysis jobs are kept.

The provenance data model used in the CBFBIRN is simplified over the HID framework’s EAV style provenance data model that was used in the FBIRN. For the CBFBIRN, the processing is centralized and under the control of the system with a predefined set of customizable processing options exposed to the end user. The provenance model is designed to capture user selected processing options and quality assessments of the processed CBF data (details are in Section “Processing Module 1: Data Upload and CBF Quantification”).

**FIGURE 2:**
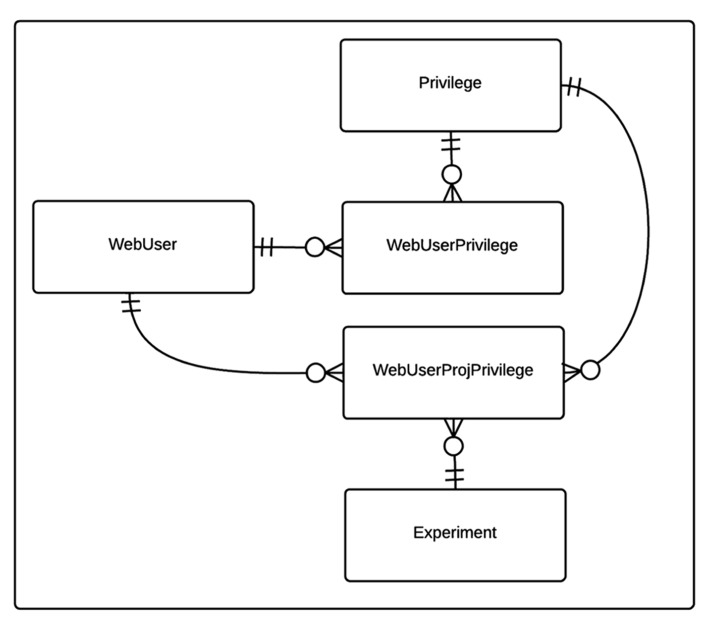
**Entity relationship (ER) diagram of CBFBIRN privilege-based security data model.** The relationships between the entities (database tables) including cardinality information are further explained in the text.

The privilege based security data model for the CBFBIRN is shown in **Figure 2**. Each user of the CBFBIRN web interface (WebUser) can have non-project specific (WebUserPrivilege) and/or project-specific privileges (WebUserProjPrivilege), regulated by the system administrator. The type of privileges available to the system is stored in the Privilege table. New privilege types can be easily added as new records to the Privilege table. Each WebUserPrivilege and WebUserProjPrivilege record has a single Privilege type. Ability to create new users and/or projects are two examples of non-project specific privileges to enable independent research groups to manage their own set of projects, giving different access privileges to their group members.

### ARCHITECTURE

The HID framework is a three tier Java 2 Platform Enterprise Edition (J2EE) system originally designed by the FBIRN to support federated clinical and imaging data management across multiple institutions. For the CBFBIRN, the HID architecture was adapted to accommodate the additional requirements of the project. First, unlike the FBIRN HID, the CBFBIRN database is a centralized system where the raw and derived data are maintained in one centralized location, which simplifies the system resource maintenance and data curation. Second, the CBFBIRN requires processing capabilities (CBF quantification processing and group analysis) in addition to the data management capability of the original HID system.

**FIGURE 3:**
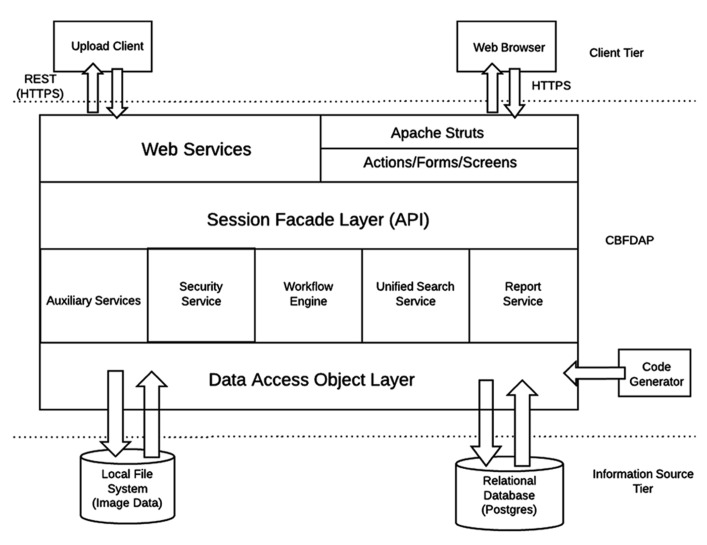
**Summary of CBFBIRN architecture**.

The overall architecture of the CBFBIRN system is depicted in **Figure 3**. Same as in Section “System Data Model,” only the added features specific to the CBFBIRN are discussed here. All user interactions, including data uploads to the system, are managed through a web browser and internet connection.

#### Workflow engine

The workflow engine manages (**Figure 3**) various workflows from two main processing modules: (1) a CBF Quantification Module that provides multiple user defined processing options; (2) a Group/Statistical Analysis Module that runs on a set of processed CBF maps derived from the first module. The workflow engine service consists of a job scheduler, a user interface (UI) to submit CBF/group analysis processing requests, and a monitoring system for the processing workflows. The job scheduler manages a job queue where all the incoming job submission requests are kept. It runs only a certain number of the job requests at a time to avoid overloading the system. It supports both one-at-a-time job submissions and batch job submissions. Since some CBF processing jobs may last more than several hours and may involve human interaction (depending on the processing options set by the user), the job scheduler provides an email notification service for important life cycle events of the workflows. The job scheduler is also programed to recover incomplete jobs in the event that the system needs to be restarted for server updates or a power outage occurs.

The job scheduler is agnostic to the workflow types it is managing, i.e., various processing paths built into both the CBF processing and group analysis modules are all easily managed by the same job scheduler. The workflow engine delegates the actual data processing needs to a stand-alone software package written in MATLAB that communicates with the job scheduler. For CBF processing, the MATLAB package also calls various AFNI and FMRIB Software Library (FSL) functions (Cox, 1996; Smith et al., 2004).

**FIGURE 4:**
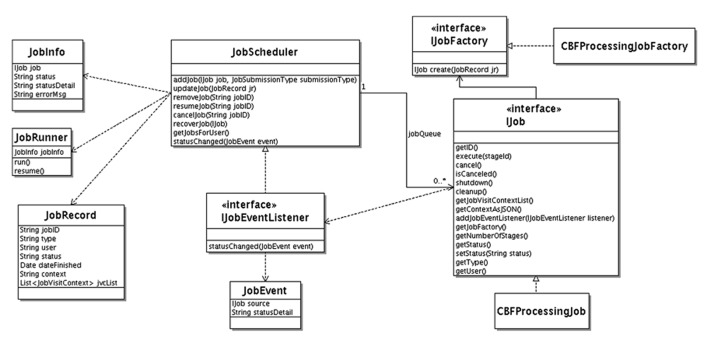
**Unified Modeling Language class diagram for CBFBIRN job scheduler**.

The Unified Modeling Language (UML) class diagram for the job scheduler component is shown in **Figure 4**. The JobScheduler class implements the workflow engine. It maintains a priority queue of Job interface implementations. Each job, implements the IJob interface. The IJob interface provides the execute() method for the core workflow functionality. In addition, it provides lifecycle methods such as cancel(), shutdown(), and cleanup() and introspection/metadata methods such as getNumberOfStages(), getContextAsJSON(), getJob-Factory().

The cancel() lifecycle method needs to be implemented for a cancelable job. This method sets a flag. The logic in execute() method needs to check this flag before starting any time consuming substep for timely job cancelation. The cleanup() method allows the job to clean up after. It is called by the job scheduler at the end of the job. The shutdown() method is called before permanent removal of the job from job queue.

A job can have a human intervention step, such as CBF job with manual ventricular annotation. The job scheduler queries the IJob interface via the getNumberOfStages() method for the number of stages of the particular job. After each stage, the job scheduler waits until the job is resumed. The user interacts with the workflows through the job management panel of the web interface from which running jobs can be canceled or waiting jobs can be resumed.

If a context is attached to the job (used for user-interaction and/or persistent jobs surviving server restart) getContextAsJSON() method returns it as string in Javascript Object Notation (JSON) representation. Jobs that require human intervention can survive server startups. These kinds of jobs can remain idle in the system sometimes for weeks until the job owner attends them. The job scheduler persists the job context for each job in the database and, uses it to revive any jobs that are still waiting for human intervention after a server maintenance incident. To accomplish this, it calls the getJobFactory() method on IJob interface, which then creates a new job instance using the passed in job context information. This method is called by the job scheduler for each revival eligible job interrupted during the server shutdown.

The job scheduler receives status updates from the jobs it manages using an event driven mechanism. It implements the IJobEventListener interface. Each job type that is interested in providing status information sends a JobEvent to IJobEventListener registered with the managed job by the job scheduler.

#### Security service

Like in the original HID, the CBFBIRN security service provides authentication and authorization services. However, the CBFBIRN security service provides finer granular (project level) privilege-based authorization services allowing the end users their own private set of projects. Within this private set of projects, a set of users with different level access privileges can be assigned. At the highest level, there is a project administrator who has full privileges to all available data. The administrator user can assign a local administrator (usually a principal investigator, PI) to the private set of projects to create and manage users for that PI’s data. Different projects for a given PI can be assigned to different users who can only see their own project. This way, multiple independent projects from multiple PIs can be supported. If the PI decides to share his/her data later, this can be easily done by the CBFBIRN project administrator by adjusting the access privileges of the other users on that particular project. This way, the CBFBIRN supports both private and shared data.

#### Reporting service

In line with the main design goal of the CBFBIRN being an end user tool, the results of CBF and group analysis workflows are accessed from a unified dynamic interface. The user can view, download results from this interface and provide data quality information on the processed data. This interface is powered by the reporting service which aggregates imaging and provenance data and provides them in a unified structure to various viewing components of the CBFBIRN.

#### Unified search service

The main disadvantage of the EAV style data model is that the queries for the data retrieval are more complex than the traditional one table per concept model (Anhoj, 2003). The main goal of the CBFBIRN search service is to provide a simple, unified, and dynamic interface for data set retrieval needs of the project. The unified search service consists of a generic web client component that the user interacts with to build a search query and a generic server side search mechanism. The user can search on two main types of the CBFBIRN metadata, i.e., clinical assessments and a host of provenance data created during different workflows. Upon user input, the web client search component builds an intermediate representation for the conditions and sends it to the server. The query integrator on the server side splits the conditions into the main queryable types (clinical assessment, provenance, quality measures), rewrites the queries and sends them to their corresponding query processor components. Each query processor converts the conditions into corresponding database queries for the relevant database tables and returns the results back to the query aggregator. Each query processor runs asynchronously. The query aggregator combines results coming from the individual query processors, ensuring that all the boolean conditions are satisfied before returning the combined result set to the user. This is accomplished by a second pass of filtering on the query processor returned data sets. All this processing is hidden from the end user who interacts with the system using a simple search interface via the CBFBIRN web.

#### Upload client

Raw CBF and supplementary image data are uploaded to the CBFBIRN database from the user’s local machine by two main mechanisms: (1) a Java WebStart application and (2) a web browser connected to the CBFBIRN web UI. While either of the two upload methods can be used, the latter method is preferred as it does not require a Java browser plugin, resulting in increased security and user convenience. The Java WebStart based upload client will be deprecated in the near future.

During the data upload process, the image header is anonymized and the data integrity check is performed. The data integrity check is important for the healthy maintenance of the database and for the minimization of unexpected post processing errors as it ensures that the corrupt and unnecessary data are excluded from the upload. During the upload process, a minimal set of demographics (age, gender, diagnosis) is also collected for each subject. Internally, the upload client communicates with the CBFBIRN system using the Representational State Transfer (REST) protocol.

### OVERVIEW OF THE CBFBIRN FUNCTIONALITY

The CBFBIRN consists of a central data repository, a PSDS, and two post processing modules. The system runs on a dedicated server (Dell Power Edge R710) located at the University of California San Diego (UCSD) Center for Functional MRI and a fully mirrored backup server located at the UCSD Supercomputer Center.

**FIGURE 5:**
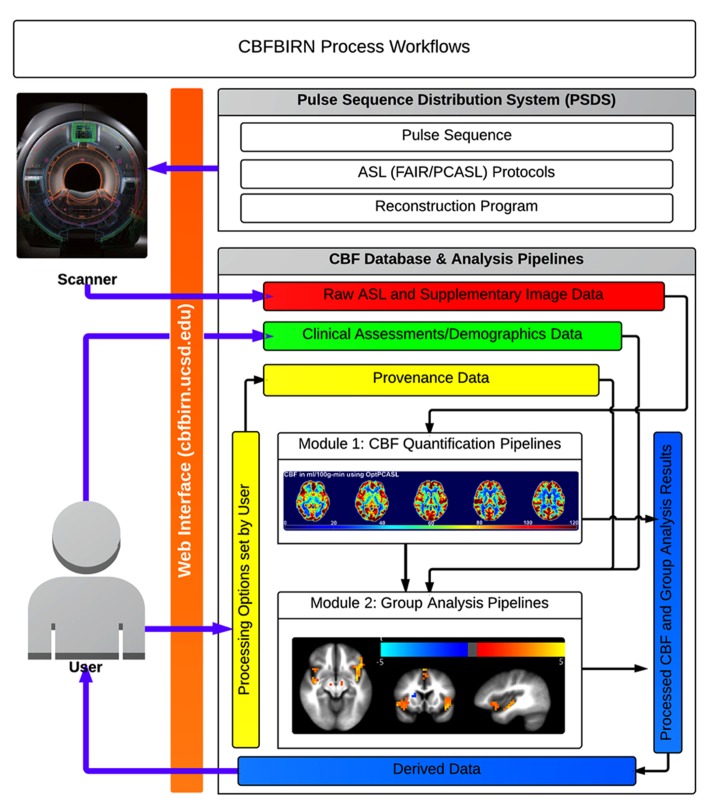
**Broad overview of CBFBIRN functionality available to end users via the web interface.** Users acquire raw ASL and supplementary image data (red box) from MR scanners using an ASL protocol downloaded from the PSDS. Data are directly uploaded to the database via the web interface. Users push these data to the CBF quantification pipeline (Module 1) to generate CBF maps, whole-brain mean gray matter CBF and additional derived data, all of which all can be downloaded (blue box) to a local computer. The database captures provenance data (yellow box), e.g., the processing options used for CBF quantification and the quality of individual CBF maps. Users contribute clinical assessments and demographics data (green box). Processed CBF maps, clinical assessments, and provenance data are fed into the group analysis pipeline (Module 2) for statistical analysis. Users can download group analysis results for further analysis and for publications.

A general overview of the CBFBIRN functionality is presented in **Figure 5**. All aspects of user interaction with the system are achieved via a web browser (orange bar) using a secure internet connection (HTTPS). Through the PSDS, users can download a MRI pulse sequence, ASL imaging protocols, and a reconstruction program as one downloadable unit, which can be used to acquire raw ASL data from GE MRI scanners (GE Healthcare Waukesha, WI). The raw data acquired from these protocols can then be uploaded to the CBFBIRN data repository using the upload mechanisms as described in the previous section. Three ASL protocols currently supported are (1) FAIR with QUIPSS II; (2) conventional PCASL; (3) multiphase pseudocontinuous ASL (MPPCASL; Jung et al., 2010).

The CBFBIRN consists of two data processing modules: (1) CBF Quantification and (2) Group Analysis Modules. The former and latter are referred to as Processing Module 1 and Module 2 in this paper.

The source code underlying the CBFBIRN database and workflows management system is available on the Neuroimaging Informatics Tools and Resource Clearinghouse (NITRC) site^2^ under the project name CBFDAP. The software is released under the Biomedical Informatics Research Network (BIRN) license and the project source files can be checked out from NITRC Subversion repository^3^. Included with the release are four separate documents, i.e., Tutorial, Developer’s Guide, Design Document, and Application Programming Interface Documentation.

We also have registered the CBFBIRN that hosts user contributed ASL and clinical data as a resource on NITRC^4^. This resource includes the project-maintained version of CBFDAP and PSDS.

In this paper, only a general description of the CBFBIRN functionality is provided. For end users, there is a detailed user manual, which is available on the project website^5^. This online documentation covers all aspects of using the CBFBIRN including (1) how to get access to the database; (2) how to upload and process data; (3) how to review and retrieve processed results. Additionally, there are several online video tutorials that guide users through important features of the CBFBIRN^6^. It should be noted that the CBFBIRN is an open-access platform, i.e., all resources and tools are open for anyone to use.

### PROCESSING MODULE 1: DATA UPLOAD AND CBF QUANTIFICATION

Module 1 of the CBFBIRN encompasses the upload of raw image data and CBF quantification processing pipeline.

#### Data upload

Data upload is handled either by a generic web browser or a Java Web Start application (**Figure 5**). For the latter, the application is downloaded from the project website and executed on user’s local machine. Written in Java, the program is OS-independent, thus able to run on Mac, PC, and Linux platforms.

The upload process guides the user to browse, select, and upload raw ASL image data in DICOM, AFNI, and GE P-file (raw K-space data) formats. If present in the path, the upload process also detects and uploads supplementary image data, which can be incorporated into the CBF quantification step. For example, (1) high resolution T1 anatomical images can be uploaded and used to create partial volume gray matter, white matter, and cerebrospinal fluid (CSF) masks using the FSL FAST program (Zhang et al., 2001), and (2) field maps can be used to correct distortions and signal dropouts in the ASL images due to magnetic field inhomogeneities (Funai et al., 2008) as an additional preprocessing step prior to CBF quantification. Users have full control over how the uploaded data are processed (see CBF Quantification).

During the upload process, the application performs data integrity checks, anonymization of DICOM headers, and automatic classification of ASL types (e.g., PCASL vs. FAIR). Supplementary data are also classified by parsing through the image header. Additionally, the subject age for a given upload is automatically determined based on the birthdate associated with the subject and the scan date extracted from the uploaded data.

#### CBF quantification

A CBF quantification job is initiated via the web interface by selecting one of the successfully uploaded subjects. Multiple jobs can also be started simultaneously if batch processing is desired.

**FIGURE 6:**
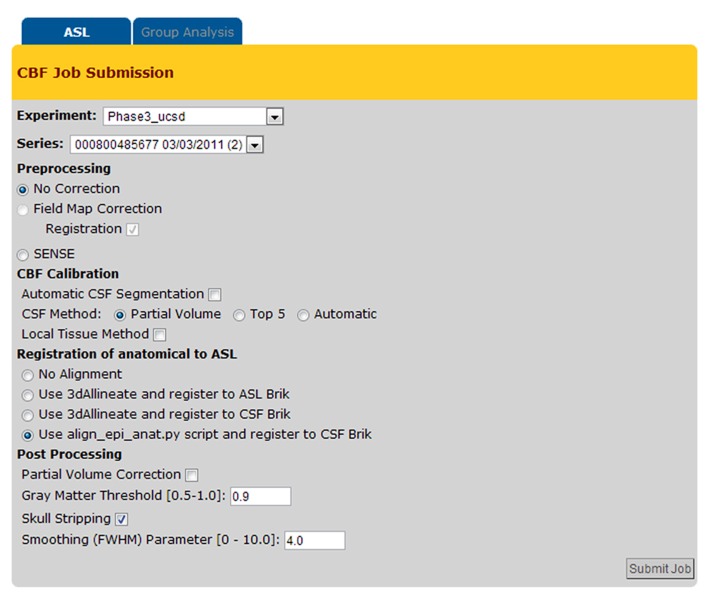
**Cerebral blood flow Job Submission page with the list of processing options**.

**Figure 6** shows a screenshot of the CBF Job Submission page that users see to select the dataset(s) and processing options for CBF quantification job(s).

The CBFBIRN automatically parses through the uploaded data, then presents the user with post processing options pertinent to the set based on availability of the supplementary image data and the acquisition type of the ASL data.

The system provides a host of CBF processing options (e.g., field map correction, partial volume correction, spatial smoothing, skull stripping, etc.), and two standard calibration methods for estimating the equilibrium magnetization of arterial blood (M_0A_), which is necessary for conversion of the perfusion signal into physiological units (ml/100g-min). The first method calculates M_0A_ using the ventricular CSF signal from a separately acquired proton density (PD) image (referred here as CSF Method in **Figure 6**; Chalela et al., 2000) while the second method (defined as Local Tissue Method in **Figure 6**) creates a voxel-wise M_0A_ map using λ, the partition coefficient, in combination with the reference PD image (Detre et al., 1992; Alsop and Detre, 1996).

After jobs are sent to the processing pipeline, the web interface provides real-time feedback on the status of each job, and sends an email notification to the user when a job is completed or if user intervention is necessary.

The CBFBIRN allows a given dataset to be processed multiple times, each time with a unique set of processing options. The multiple processing feature is particularly useful for comparing the effects of different options on the CBF quantification process and allows users to find the best parameter sets for their particular data. Using the provenance data stored in the CBFBIRN for each job, the system tries to minimize unnecessary repetition of long running tasks such as field map processing by using existing field map results in subsequent runs where field map analysis is required. Since field map correction is the most time consuming step of the CBF processing (up to 90% of the processing time for field map correction of MRI data acquired with non-Cartesian trajectories), this optimization greatly decreases the processing time of subsequent runs on the same data set. For a given dataset that has been processed multiple times, the final results are aggregated and presented in the Processing Summary Page (see **Figure 10**).

Via the Processing Summary Page, users can also review the CBF maps and detailed process logs, as well as download the processed results to a local storage unit (see also **Figure 9**). Additionally, the user can assign a qualitative rating score to each of the processed CBF maps. The rating entered is stored in the database as provenance data, which is used as a filter criterion in selecting a subset of processed jobs for a group analysis. For example, users can exclude a set of CBF maps that do not meet a quality standard (e.g., those labeled as “Marginal” and/or “Unusable”) from a group analysis.

### PROCESSING MODULE 2: GROUP ANALYSIS

**FIGURE 7:**
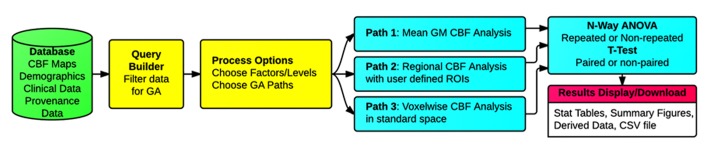
**Schematic of the group analysis workflows, consisting of (1) Filtering of CBF maps from the database (Query Builder in yellow); (2) Selecting the number of factor(s) and corresponding factor levels (Processing Options in yellow); (3) Choosing one of the three types of group analysis supported (Paths 1, 2, and 3 in cyan); (4) Reviewing of group analysis results (*n*-way ANOVA/*t*-test in cyan), (5) Reviewing and downloading processed results (Results Display/Download in pink)**.

Three types of group analysis are supported by the CBFBIRN. **Figure 7** shows a schematic of the workflows associated with the CBFBIRN group analysis. The workflows can be broadly broken into five steps, i.e., (1) Choosing a subset of CBF processed maps using a built-in query builder; (2) Selecting factors and associated factor levels for each factor; (3) Choosing one of the three group analysis paths; (4) Reviewing of group analysis results in the form of statistical analysis tables and plots; (5) Downloading of processed data for storage and for further analysis offline.

#### Data filtering for group analysis

**FIGURE 8:**
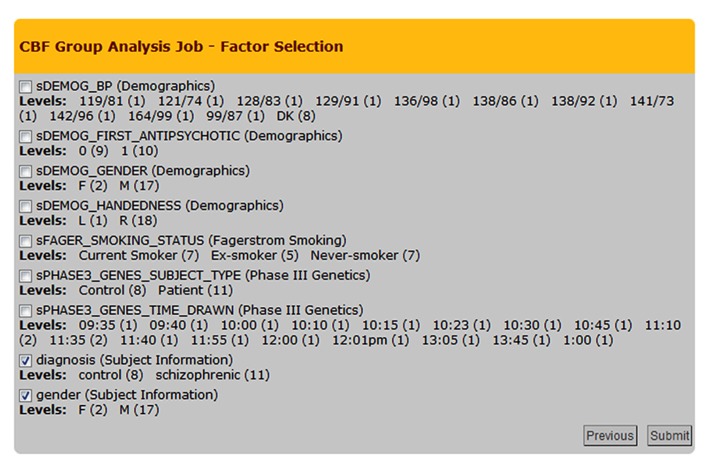
**Cerebral blood flow Group Analysis Job – Factor Selection page from an example group analysis job.** Nine factors and corresponding factor levels are shown based on the user-selected CBF maps, in which two factors are selected, i.e., Diagnosis and Gender.

The group analysis begins with a selection of the processed CBF maps. The built-in query builder uses several search attributes including data provenance (e.g., CBF quantification options used, quality of the CBF maps), and subject demographics/clinical assessments. This is schematically shown in **Figure 5** with the three arrows feeding the Processing Module 2: Group Analysis Pipelines box. Based on a given search, the system locates the CBF maps and associated demographics/clinical assessments and presents the user with factors and factor levels associated with the selection. Gender and diagnosis are examples of factors and their corresponding factor levels are male/female and controls/schizophrenic patients, respectively. **Figure 8** shows a screenshot of the Group Analysis page from a representative study where factor and factor level selections are made.

#### Group analysis processing paths

Three types of group analysis are supported by the CBFBIRN, Path 1: whole-brain mean gray matter analysis; Path 2: regional analysis for each of the user defined region of interests (ROIs); and Path 3: voxel-wise standard space analysis (**Figure 7**).

As the name implies, Path 1 performs a statistical analysis for a group of user-selected CBF maps using the whole-brain mean gray matter CBF value calculated from each subject as the dependent variable.

For Path 2 ROI-based analysis, users upload subject-specific ROI files and associate each file with a specific subject in the database via the web interface. The project website provides a stand-alone wrapper script written in MATLAB^7^ that users can download to generate the subject-specific ROI files from individual anatomical images on their local computer. The script calls the FreeSurfer ASEG program, which performs automatic cortical and subcortical segmentation (Fischl et al., 2002). The script generates a ROI file for each subject in AFNI BRIK format, containing cortical and subcortical regions labeled by unique integer numbers.

Using the filtered CBF maps and the associated ROI files, the Path 2 group analysis pipeline performs an appropriate statistical test for each and every region individually as identified by the ROI files.

For Path 3, the system first warps individual CBF maps to the standard space using the anatomical data as the base template and then performs an appropriate statistical test on a per-voxel basis.

For all of the processing paths, the CBFBIRN generates an intermediate comma-separated value (CSV) file containing CBF measures and other associated assessments/demographics, and a statistical test is performed using this file.

The type of statistical test invoked for a specific group analysis job is automatically determined by the CBFBIRN based on the factor/factor levels and the type of group analysis path selected (**Figure 8**). The system also determines whether a requested job has a repeated measures design by analyzing the structure of the input data set, in which case either a paired *t*-test or a repeated measures analysis of variance (ANOVA) is performed depending on the number of factors selected.

## RESULTS

### PROCESSING MODULE 1: DATA UPLOAD AND CBF QUANTIFICATION

#### Reviewing of processed CBF data

**FIGURE 9:**
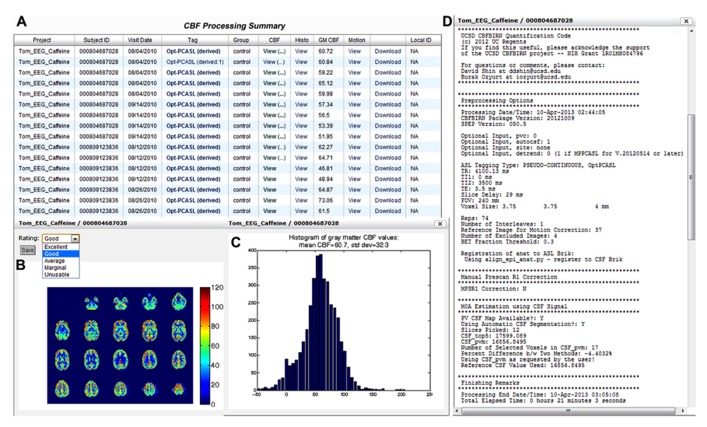
**Cerebral blood flow Processing Summary Page that presents a table (A)** containing the complete list of successfully processed jobs down the rows. For each job, the table provides useful details such as the subject ID, scan date, experimental condition, ASL protocol, and whole-brain mean gray matter CBF value. A CBF map **(B)**, a histogram of gray matter CBF values **(C)**, and detailed processing logs **(D)** are shown from a representative job, all of which are accessible directly from the table.

**Figure 9A** shows a screenshot of the CBF Processing Summary Page from a representative project. The page contains a table where each CBF job processed is shown as a separate row. The quantified CBF map in the physiological units (ml/100g-min), histogram of gray matter CBF values, and motion parameter plots are accessible by clicking on the corresponding cell in the table (Figures 9B,C). When a pop-up window containing the CBF map is presented, the user can assign a quality rating, which is stored as the provenance data. The user can also download the processed results by clicking the Download link available directly from the table. Additionally, the table lists the project name, CBFBIRN assigned subject ID, scan date, experimental condition, the type of ASL protocol used, and the mean gray matter CBF value. By clicking on the ASL protocol name (Tag column), the user can view detailed provenance information for each job, including input parameters for the CBF processing, as well as processing date, time, and duration (**Figure 9D**).

#### Multiple processing support

**FIGURE 10:**
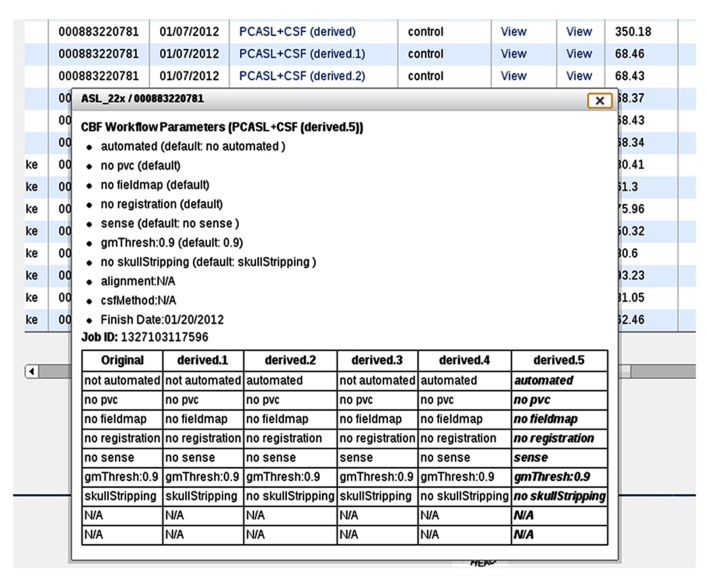
**A processing log for an example data set for which the CBF processing was done six times, each with a unique set of CBF processing options.** The table at the bottom lists all jobs and processing options used for each, allowing the user to easily compare differences between them.

In the event that the same CBF data set is processed multiple times with different input parameters, additional information is presented in the processing logs (**Figure 9D**), specifically, a table listing provenance data for each processing run for easy input parameter difference comparisons (**Figure 10**). Each run on the same data set is uniquely labeled, e.g., original, derived, derived.1, derived.2, etc.

### PROCESSING MODULE 2: GROUP ANALYSIS

**FIGURE 11:**
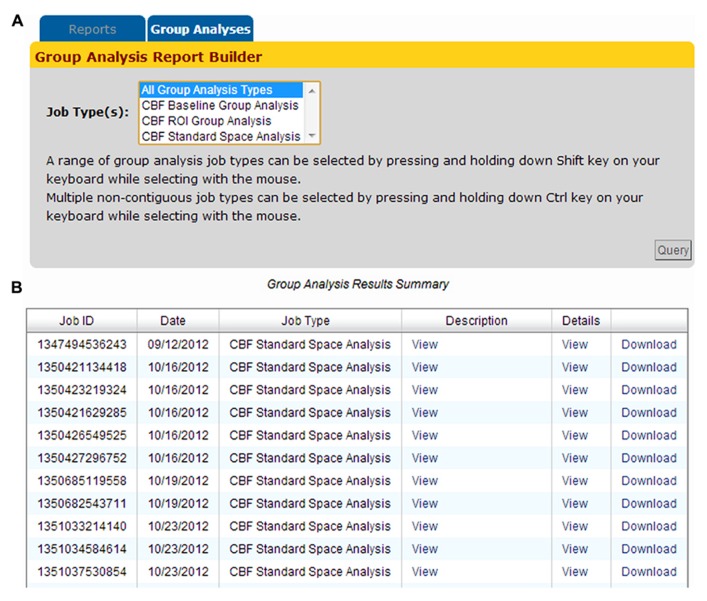
**(A)** Group Analysis Report Builder page used to define the type(s) of processed jobs to be displayed on the Group Analysis Results Summary table. **(B)** The table provides a quick access to the relevant attributes of processed jobs including the Job ID, processing date, the type of group analysis performed, and a pop-up window containing a summary of the statistical analysis tables and graphs.

Group analysis jobs processed by the CBFBIRN are grouped into three categories (Baseline, ROI, and Standard Space) as shown in **Figure 11A**. Adopting the same presentation style implemented to display CBF processing jobs (previous section), all group analysis jobs are listed in the Group Analysis Results Summary table (**Figure 11B**). Each row contains useful attributes for each job, including the CBFBIRN-generated Job ID, processing date, the type of group analysis performed, and a detailed report that contains the summary of the statistical analysis results. The figure also shows the Download link that allows users to download statistical graphs in Portable Network Graphics (png) file format and a CSV file containing the summary data used to perform the relevant statistical tests.

#### Group analysis Path 1: whole-brain mean gray matter CBF analysis

**FIGURE 12:**
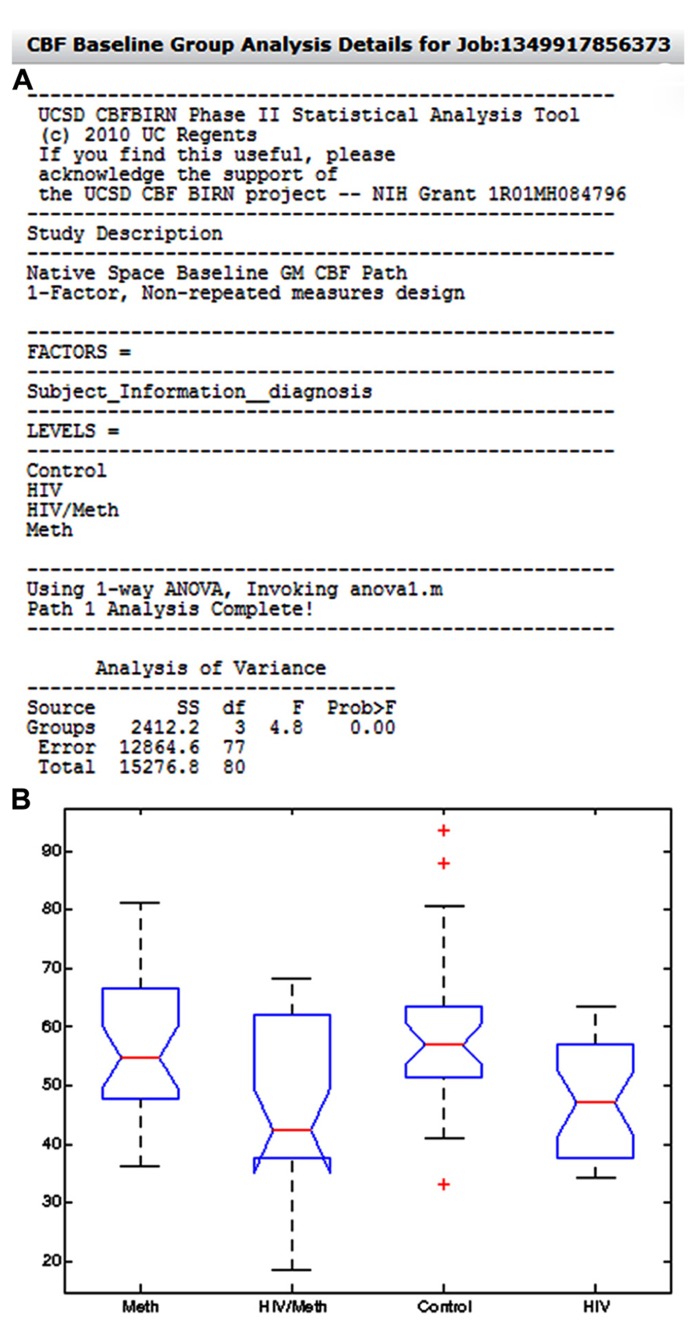
**(A)** The system-generated group analysis report from an example study, looking at the effect of HIV and methamphetamine use on whole-brain gray matter CBF. Also shown in the report is a box plot **(B)** summarizing the range and mean of gray matter CBF values across factor levels.

**Figure 12A** shows an example group analysis result processed by the whole-brain gray matter CBF analysis path and as presented in the Group Analysis Results Summary table. The report shows the user selected factor (Diagnosis), the corresponding factor levels (Control, HIV, Meth, HIV/Meth), and the statistical test invoked by the CBFBIRN (one-way ANOVA). Additionally, the report contains the ANOVA table as well as the box plot summarizing the range and mean of gray matter CBF values for each factor level (**Figure 12B**).

**FIGURE 13:**
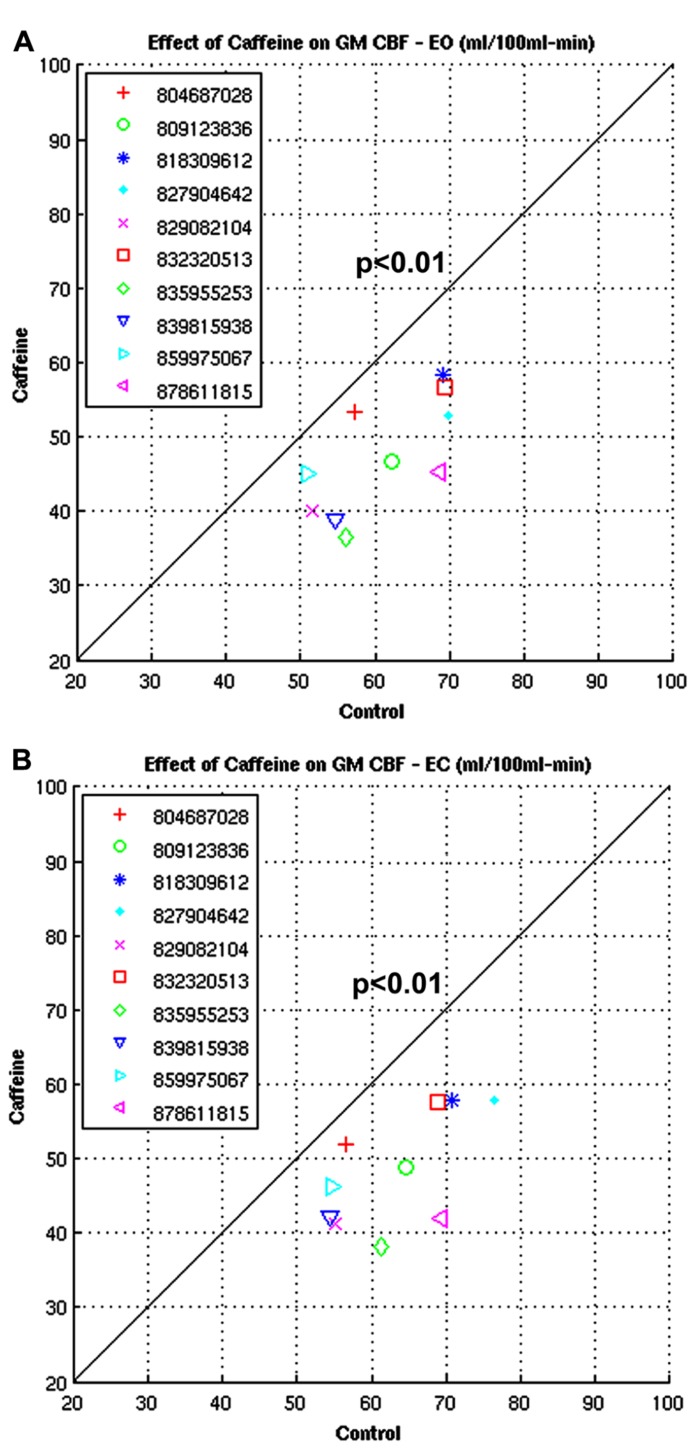
**(A,B)** Plots from a study looking at the whole-brain gray matter CBF measures before and after caffeine consumption involving 10 healthy volunteers under EO and EC conditions.

Figures 13A,B show the CBFBIRN-generated graphs from another group analysis. The results came from a study looking at the effect of caffeine consumption on whole-brain gray matter CBF involving 10 healthy volunteers. Shown are significant reductions in gray matter CBF both in eyes-open (EO; **Figure 13A**) and eyes-closed (EC; **Figure 13B**) conditions. The system invoked a paired *t*-test after automatically detecting that the study had a repeated measures design.

#### Group analysis Path 2: regional CBF analysis

**FIGURE 14:**
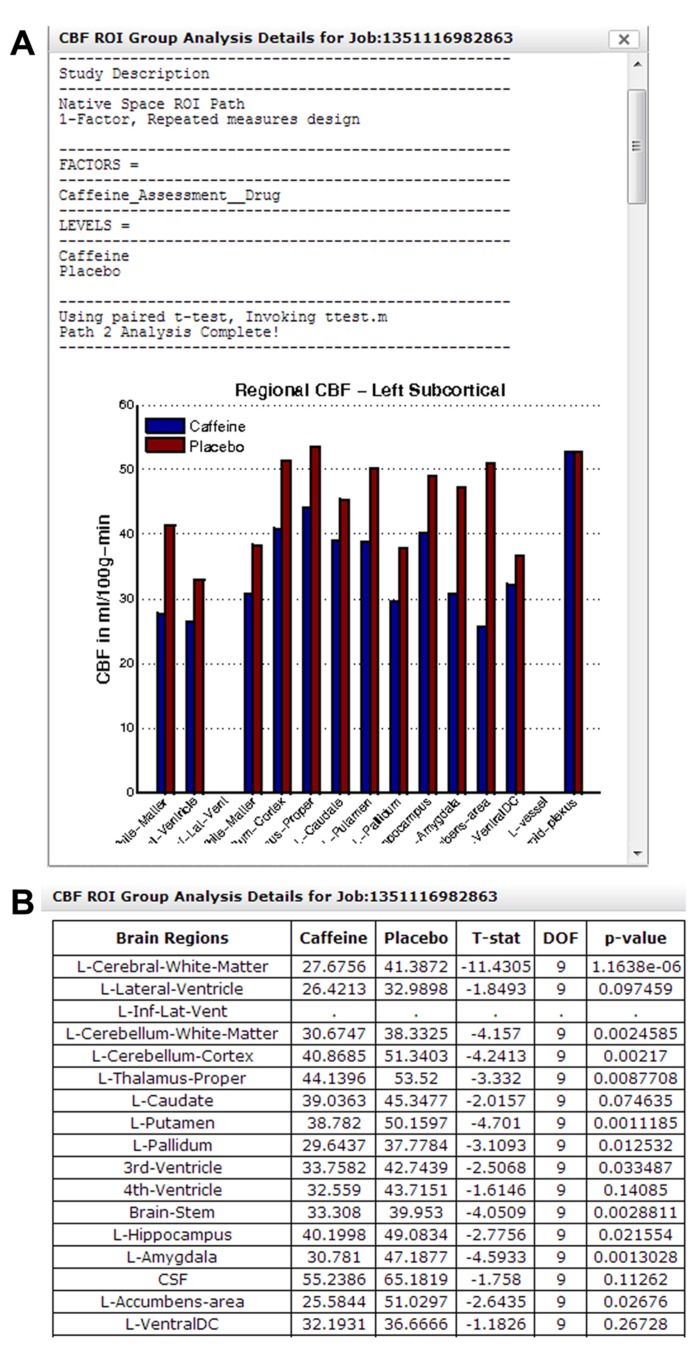
**(A)** A section of the group analysis report from a caffeine study processed using Group Analysis Path 2. The graph at the bottom shows a widespread reduction in CBF for all regions after caffeine consumption. **(B)** A section of the system-generated CSV file, summarizing CBF changes in each brain region and associated paired *t*-test result/*p*-value.

**Figure 14A** shows a section of the group analysis report generated by the regional CBF analysis pipeline. The result shows a decrease in CBF for all regions shown with caffeine consumption. Note that results from Figures 13 and 14 came from the same study, but were processed in two different group analysis paths. **Figure 14B** shows a section of the downloadable CSV file displayed in the same group analysis report.

#### Group analysis Path 3: voxel-wise standard space analysis

**FIGURE 15:**
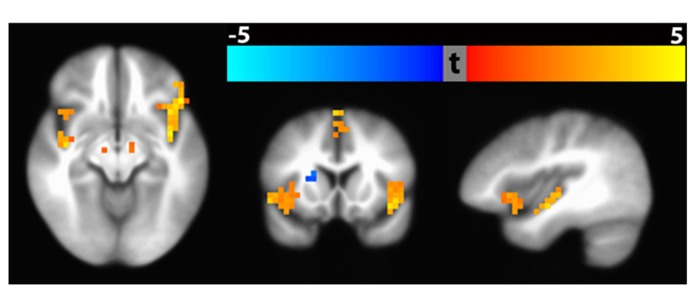
**Significant clusters (*p* < 0. 01, corrected) based on baseline CBF difference between schizophrenic and healthy subjects using Path 3 Voxel-wise Standard Space Analysis.** Bilateral clusters in the inferior frontal gyrus and parahippocampal gyrus are shown in the axial slice (hypoperfusion with Schizophrenia). The coronal slice shows hypoperfusion in the inferior frontal gyrus and hyperperfusion in the right putamen. The sagittal slice shows hypoperfusion in the anterior and posterior insula.

An example result from the voxel-wise group analysis path is shown in **Figure 15**. The figure shows regional differences in baseline CBF between healthy (*n* = 112) vs. schizophrenic subjects (*n* = 122). The result identified 13 significant clusters (*p* < 0.01, corrected) including bilateral inferior frontal gyrus extending to anterior insula, medical frontal gyrus extending to anterior cingulate gyrus, superior frontal gyrus extended to cingulate gyrus, parahippocampal gyrus as well as left superior temporal gyrus extending to posterior insula. In the colorbar, orange denotes higher CBF in control subjects and blue denotes higher CBF in schizophrenic patients. The raw ASL data and clinical assessments were contributed from the multi-site FBIRN Phase 3 study (Ford et al., 2009; Potkin and Ford, 2009; Potkin et al., 2009; Shin et al., 2013) and processed by the CBFBIRN.

## DISCUSSION

The CBFBIRN capabilities described in this paper support many aspects of conducting an ASL study, including (1) acquisition of raw data using the ASL protocols provided by the PSDS; (2) uploading of raw data to the CBFBIRN database; (3) post processing that produces quantified CBF maps; and (4) performing statistical analysis through the CBFBIRN group analysis pipeline.

With public release of the source code of the CBFBIRN system framework via NITRC, neuroinformatics researchers can not only replicate the system we have implemented, but they can also adapt and extend it for many applications where a web-based database with data management and processing capabilities are desirable. While we used the system to promote storing, processing and sharing of ASL data, the system can be extended to handle virtually all types of scientific data. Given that there is a growing trend and effort for data sharing across many disciplines of scientific research, the system infrastructure presented here may be a useful resource.

Aside from data sharing, the CBFBIRN promotes standardization of data acquisition and processing, which is particularly timely for the ASL community. Recently, the ISMRM Perfusion Study Group issued the first white paper with recommended data acquisition and processing guidelines for clinical applications, citing that the “overabundance of choices is an impediment to the acceptance of ASL by the clinical community, complicating the implementation of ASL in standard care, comparisons between sites and the establishment of meaningful clinical trials.” The CBFBIRN data sharing and processing capabilities dovetail well with this concerted effort toward standardization. The efforts of the Perfusion Study Group, as well as our own, can help accelerate the rate of adoption of ASL by researchers and clinicians.

The CBFBIRN provides a broad range of CBF processing options including two widely used calibration methods for conversion of the perfusion signal into physiological units (ml/100g-min; Detre et al., 1992; Alsop and Detre, 1996; Chalela et al., 2000). These processing options provide users flexibility and choices over how their data are analyzed. With the multiple processing capability (**Figure 10**), the CBFBIRN allows efficient comparison of CBF maps processed with different set of options. Given these features, the CBFBIRN can be used as a testbed for evaluating a new processing method on existing data in the database, prior to its integration into the CBFBIRN processing pipeline for general use. Additionally, the overhead associated with managing and processing a large number of data sets is minimized by taking advantage of the batch processing capability of the CBFBIRN (**Figure 6**).

Aside from the task of acquiring and processing the collected data, researchers often devote a large amount of time and resources toward archiving both raw and processed data, compiling them into a format suitable for statistical analysis, and running the actual statistical analysis itself. Additionally, different types of statistical analysis oftentimes require a new round of data formatting and programing scripts. The preprocessing steps and statistical analysis are handled and executed by the CBFBIRN and abstracted from the user, reducing the user task to initiation of the group analysis job and receiving of an email notification at the job completion. In the event that an additional analysis is needed, users can pursue that offline with the system-generated CSV file optimally formatted for importing into commercial statistical packages.

Current users have reported that the CBFBIRN greatly reduces the time it takes from data acquisition to reporting of study findings. Since its introduction to the research community in 2011, several investigators have published studies that have used the CBFBIRN as a primary tool for data storage and processing (Ho et al., 2012, 2013; Wierenga et al., 2012; Shin et al., 2013).

One crucial component that makes group analysis possible on the CBFBIRN workflow is the advanced query builder that provides flexibility in formulating hypotheses for statistical testing. The query builder aggregates all forms of data available in the database, including provenance data (e.g., quality assessment of CBF maps, set of processing methods invoked) and clinical assessments (e.g., Caffeine vs. Placebo, Schizophrenia vs. Control, Old vs. Young, Men vs. Women) and employs them as search criteria, allowing users to create a set of CBF maps for testing a specific hypothesis. For example, it is straightforward to search and select data corresponding to male schizophrenic patients aged 30 or above and from gender/age matched healthy controls with the additional constraint of excluding CBF maps rated as “unusable” to investigate CBF differences between these two groups.

While the post processing workflows described in this paper are tailored for raw data acquired from the scan protocols provided by the CBFBIRN PSDS, the CBFBIRN workflows can be extended to accommodate additional data formats that are supported by other ASL research groups. Integration of these data allows other groups to benefit from the CBFBIRN system infrastructure and capabilities. Hundreds of ASL scans from the previous FBIRN studies have already been integrated into the CBFBIRN with minor adjustment to the processing program, including those acquired from another MRI vendor having a different data format.

To date, the CBFBIRN hosts more than 1,300 data sets from 22 different projects and the storage capacity can accommodate several times the current data size. Some of the experimental conditions under which the ASL were collected include Alzheimer’s disease, schizophrenia, bipolar disorder, depression, traumatic brain injury, HIV, caffeine usage, and methamphetamine abuse.

All data currently stored in our database have been contributed by research studies reviewed and approved by the institutional review board (IRB) at their institutions. Since the primary role of this project is in building and providing a platform for data sharing, our general policy is that it is the responsibility of users to ensure that the data they contribute on the CBFBIRN are collected according to the IRB standards. However, platform providers can play an important role in promoting IRB compliance by requiring all users to submit a copy of the IRB form for review before they can use their system resources. Our project has this requirement in place for new users.

### DATA SHARING ISSUES

The timing and quantity of the data shared for public access is at the discretion of each project’s PI, though we do stipulate that all data is to be shared one year after the project end date. Currently, most users rely on the CBFBIRN for storing and processing new data for ongoing studies. These data are visible only to the PI and designated users, and only the PI has the full authority to make the data public. Once public access is granted by the PI, the modification of access privileges is made at the CBFBIRN administrative level (**Figure 2**). The publically accessible data from previous FBIRN studies allow new users with the opportunity to test-drive the CBFBIRN. As the project progresses and the PIs from ongoing studies complete data collection, analysis, and publications, we expect more data to be available for public access in the future.

We have seen a moderate adoption of the CBFBIRN by the research community since its introduction in 2011, particularly with availability of the ASL protocols via the CBFBIRN PSDS. However, adoption of the CBFBIRN by clinicians has been slow. Based on our experience, the primary concerns for data sharing revolve around maintaining the privacy of subject/patient information as well as misgivings about sharing valuable data in a public data repository prior to a group’s publication although we anonymize subject information during data upload and never share data publicly without the explicit permission and consent of the PI. Ultimately, the viewpoint of the scientific community in regards to data sharing must be fundamentally changed. The success of this cultural shift will be heavily determined by the efforts of federal funding agencies such as the NIH and research consortiums like the ASL Study Group.

### LESSONS LEARNED

Building a large-scale database requires clear identification of all the features that will be available to end users and the selection of the system model and architecture that will support them. Planning with an end in sight is crucial in order to avoid wasted time and resources. In the case of the CBFBIRN, for example, the ability to accommodate an unlimited number of clinical assessments and associating them to subject-specific raw ASL data was an important requirement in order to offer end users the ability to perform statistical analysis. Clinical assessment scores exist in different data types (string, integer, real number, etc.) and each assessment may contain multiple sublevels and score items. In order to accommodate this, we chose an EVA style database design, which allowed system extension without database schema changes, i.e., system extension was achieved by storing raw data and metadata in the same schema.

Another aspect of implementing a large scale data management platform that requires a careful initial planning is UI design. While an intuitive and easy to use UI is critical for its success, this task becomes exceedingly difficult as the number of capabilities available to users increases. From our experience, a careful design of the UI at an early phase of the system implementation helped avoid major modifications that can be time consuming later on. For the CBFBIRN, all user interactions take place via a web browser through a UI that accommodates all functionality, including data upload, addition and association of clinical assessments to existing data, CBF processing, group analysis, and the ability to download and share data.

Also important for the long-term viability of an online database is implementation of an automated and stringent data inspection mechanism during data upload. For the CBFBIRN, the system filter reviews all files that users attempt to upload and allows only those relevant for the system, eliminating the accumulation of unnecessary data in the database. For the relevant files, the filter also checks for data integrity to minimize potential errors on the processing workflows.

In conclusion, the CBFBIRN is an open-access online platform that supports data storage, processing, and sharing of image data. While it is designed to support the needs of the ASL community for CBF quantification processing, group analysis, and data sharing, the system architecture can be extended to include additional types of data, such as resting-state functional magnetic resonance imaging (fMRI) data. We have demonstrated its utility, ease of use, data security, respect for intellectual property, and compliance to HIPAA requirements. Ultimately, the full potential of the CBFBIRN will be realized by the active participation of the neuroimaging community in the process of contributing and sharing their data.

## Conflict of Interest Statement

The authors declare that the research was conducted in the absence of any commercial or financial relationships that could be construed as a potential conflict of interest.

## Footnotes

1

2

3

4

5

6

7
